# *Pparg* may Promote Chemosensitivity of Hypopharyngeal Squamous Cell Carcinoma

**DOI:** 10.1155/2020/6452182

**Published:** 2020-04-22

**Authors:** Meng Lian, Jiaming Chen, Xixi Shen, Lizhen Hou, Jugao Fang

**Affiliations:** Department of Otorhinolaryngology Head and Neck Surgery, Beijing Tongren Hospital, Capital Medical University, Beijing 100730, China

## Abstract

The upregulation of peroxisome proliferator-activated receptor gamma (*PPARG*) has been shown to increase the chemosensitivity of several human cancers. This study is aimed at studying if *PPARG* sensitizes hypopharyngeal squamous cell carcinoma (HSCC) in chemotherapeutic treatments and at dissecting possible mechanisms of observed effects. We integrated large-scale literature data and HSCC gene expression data to identify regulatory pathways that link *PPARG* and chemosensitivity in HSCC. Expression levels of molecules within the *PPARG* regulatory pathways were compared in 21 patients that underwent chemotherapy for primary HSCC, including 12 chemotherapy-sensitive patients (CSP) and 9 chemotherapy-nonsensitive patients (CNSP). In the CPS group, expression levels of *PPARG* were higher than that in the CNSP group (log‐fold‐change = 0.50). Structured text mining identified two chemosensitivity-related regulatory pathways driven by *PPARG*. In the CSP group, expression levels for 7 chemosensitivity-promoting genes were increased, while for 13 chemosensitivity suppressing the gene expression levels were decreased. Our results support the chemosensitivity-promoting role of *PPARG* in HSCC tumor cells, most likely by affecting both cell proliferation and cell motility pathways.

## 1. Introduction

As one of the most common head and neck tumors, hypopharyngeal squamous cell carcinoma (HSCC) accounts for more than 160,000 new cases and 83,000 deaths annually [1]. In Europe and the United States, HSCC has been ranked as one of the most common human malignancies [2]. High risks of metastasis to cervical lymph nodes and a lack of evident clinical symptoms make it a challenge for the diagnosis and treatment of HSCC [3]. Novel therapies for HSCC are warranted.

PPARG gene encodes a member of the peroxisome proliferator-activated receptor (PPAR) subfamily of nuclear receptors. Several previous studies suggested that the upregulation of *PPARG* might induce chemosensitivity in human carcinomas [4–6]. In particular, in human nonsmall-cell lung carcinoma cells, the activation of PPAR*γ* is capable of overcoming the NRF2-dependent chemoresistance [4]. In basal-like breast carcinoma, *PPARG* activation significantly reduces the expression of MnSOD and increases chemosensitivity [5]. In turn, the silencing of *PPARG* decreases the chemosensitivity of pancreatic cancer cells in vitro [6]. So far, the involvement of PPARG was explored in some but not other squamous cell carcinoma with location in the head or neck, with the emphasis being made on oral cancer. For example, in cell lines of oral carcinoma origin, the treatment with a synthetic retinoid, 4-hydroxyphenylretinamide was shown to result in an increase of HSCC chemosensitivity [7]. PPARG has been suggested as a target for chemoprevention in head and neck cancer prevention, which was based on consistent evidence from investigations of human tumor cell line studies, epidemiological analysis, and animal carcinogenesis models [8]. PPARG has also been suggested as a potential therapeutic target gene for oral squamous cell carcinoma [9], and the activation of PPARG was shown to downregulate several features of the neoplastic phenotype in human upper aerodigestive tract tumors [10].

In this study, we hypothesize that PPARG may play roles in HSCC chemotherapy by influencing the tumor cell chemosensitivity. To test this hypothesis, we collected expression profiles from primary HSCC patients that underwent chemotherapy and tested the PPARG expression variations between chemotherapy-sensitive patients (CSP) and chemotherapy-nonsensitive patient (CNSP) groups. Then, we used large-scale literature data mining to identify molecular pathways that are driven by PPARG to influence the chemotherapeutical activities within the HSCC patients. Our results suggested that the increased expression of PPARG might promote the chemosensitivity of HSCC cells through multiple molecular pathways.

## 2. Materials and Methods

### 2.1. Patient Recruit and Specimen Selection

Twenty-one HSCC patients were recruited by the Department of Head and Neck Surgery, Beijing Tongren Hospital, including 12 chemotherapy-sensitive patients (CSP) and nine chemotherapy-nonsensitive patients (CNSP). All patients received two-periodic chemotherapies induced by TPF (taxane/cisplatin/5-FU). Tissue specimens were collected from each of these patients after resection during surgery. Each sample was immediately snap-frozen in liquid nitrogen and stored at -80°C. This study has been approved by the ethics committee of Beijing Tongren Hospital, and written agreement has been acquired from each participant.

### 2.2. RNA Extraction, cDNA Synthesis, and In Vitro Transcription

mRNA was extracted from tissue samples using TRIzol (Invitrogen), and then, RNA quantity was examined by denaturing gel electrophoresis, which revealed at least two distinct bands representing 28S and 18S ribosomal RNA, suggesting no DNA contamination or RNA degradation. First, reverse transcription was used to synthesize the first-strand cDNA, and second-strand cDNA synthesis was used to convert single-stranded cDNA into double-stranded DNA with a PrimeScript™ Double Strand cDNA Synthesis Kit (TAKARA). Second, after purification by removing RNA, primers, enzymes, etc., the double-stranded DNA was used as a template for the transcription of biotinylated cRNA in vitro. Finally, the biotinylated cRNA was purified and prepared for hybridization with a prepared microarray.

### 2.3. Acquirement of mRNA Expression Profiles of HSCC

For the microarray mRNA expression profile of HSCC, the Illumina Human HT-12 Bead Chip was applied for hybridization with the labeled cRNA. There are six types of internal parameters and 887 probes in this microarray for the quality control of all samples. Briefly, the cRNA samples were hatched with the Illumina Human HT-12 Bead Chip at room temperature and subjected to high-temperature washes, ethanol washes, and three washes at room temperature. After desiccation, images were collected with the Illumina Bead Chip Reader software. Illumina Genome studio-Gene Expression software was employed to filter background noise and the missing value effect in the raw data. The quantile method was used for normalization. The gene expression profile was obtained using Illumina Custom software.

### 2.4. Literature-Based Pathway Analysis

Large-scale literature-based functional pathway analysis was performed to investigate potential biological associations between PPARG and chemosensitivity and constructing PPARG regulatory pathways. We first identified PPARG downstream molecular targets with polarity; then, we explored the chemosensitivity upstream positive and negative regulators. Each relationship (PPARG-gene and chemosensitivity-gene relationship) has at least one reference, which is covered by Pathway Studio (https://www.pathwaystudio.com). We provided detailed information about each relationship in PPARG_HSCC→Regulatory_Pathway1 and Regulatory_Pathway2, including the types of associations, the number of underlying supporting references, and the sentences where these associations had been identified and described.

Then, we tested that expression changes of the molecules involved in the pathway to associate it with HSCC by using HSCC expression data. The expression changes of PPARG and the 20 genes involved in the PPARG-chemosensitivity regulatory pathway were compared between CSP and CNSP groups.

### 2.5. Gene Set Enrichment Analysis (GSEA)

To test the molecular functions of the identified PPARG driven triggers that regulate the chemosensitivity in HSCC, a GSEA was conducted for each PPARG-chemosensitivity regulatory pathway; results were reported and compared.

## 3. Results

### 3.1. PPARG Expression in CSP Group

**Figure 1:**
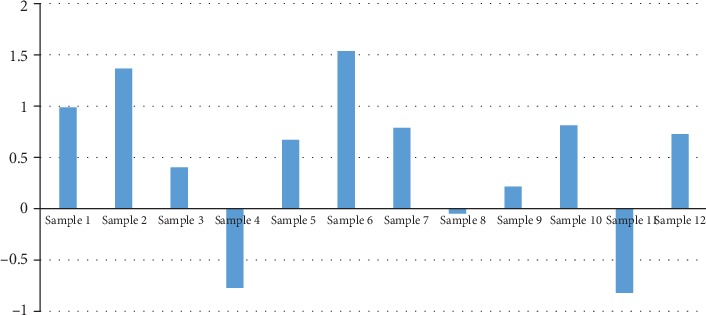
PPARG expression in terms of log‐fold‐change (LFC) in chemotherapy-sensitive patients (CSP) among each of the 12 samples.

We presented the expression of PPARG for the 12 chemotherapy-sensitive HDCC patients in Figure 1. Overall, PPARG demonstrated an average 40% increase in the CSP group (LFC = 0.50). Nine out of the 12 patients presented increased expression levels compared to the CNSP group. However, there was a significant variance among the individuals within the CSP group (std = 0.75), with three of them demonstrated expression, resulting in a nonsignificant *p* value = 0.25. These results suggested that PPARG might not be the only factor that influences the chemotherapy sensitivity of HDCC patients.

### 3.2. PPARG Stimulates Chemosensitivity Promoters

As shown in Figure 1, literature-based data mining identified seven chemosensitivity promoters that have been promoted by PPARG. The expression of these molecules, including PPARG, was upregulated in the CSP group, which supports the pathway identified from the literature knowledge database. For the 54 references supporting the pathway given in Figure 1, please refer to PPARG_HSCC⟶Regulatory_Pathway1. For the expression levels of the eight molecules in Figure 1, please refer to PPARG_HSCC⟶Expression_LFC.

To explore the functionality of the molecules included in the chemosensitivity-promoting pathway presented in Figure 1, a GSEA has been conducted using against the Gene Ontology (GO). The top 10 GO terms enriched were presented in Table 1. More detailed information of the 12 enriched GO terms passed false discovery ratio (FDR) analysis (*q* = 0.05) was presented in PPARG_HSCC⟶GSEA1. From Table 1, it can be seen that the genes were mainly related to cell proliferation and cell apoptotic, suggesting that PPARG may promote the chemosensitivity through the regulation of cell proliferation-related pathways. To note, PPARG directly plays roles in 6 out of 10 of these GO terms.

### 3.3. PPARG Suppresses Chemosensitivity Inhibitors

**Figure 2:**
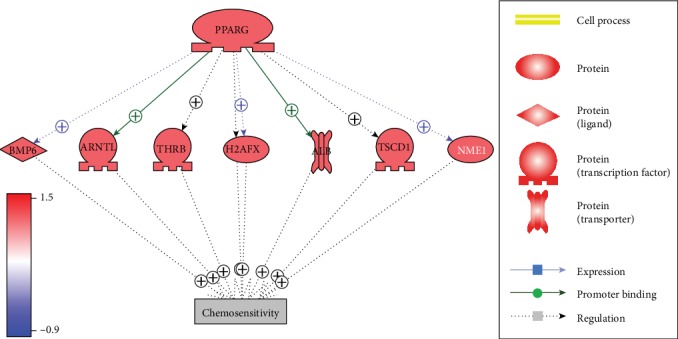
The chemosensitivity-promoting pathway built through literature data analysis and tested by HSCC expression data. The red color of genes represents increased expression in the chemosensitive group compared with the nonchemosensitive group.

As shown in Figure 1, literature-based data mining identified 14 chemosensitivity inhibitors that have been suppressed by PPARG. The expression of these molecules was downregulated in the CSP group, which supports the pathway identified from the literature knowledge database. For the 172 references supporting the pathway given in Figure 2, please refer to PPARG_HSCC⟶ Regulatory_Pathway2. For the expression levels of the eight molecules in Figure 2, please refer to PPARG_HSCC⟶Expression_LFC.

GSEA results showed that the GO terms enriched by the 15 genes (including PPARG) were mostly related to the regulation of cell motility and locomotion (see Table 2). The top 10 GO terms enriched were presented in Table 2. More detailed information of the 65 significantly enriched GO terms (FDR = 0.05) was presented in PPARG_HSCC⟶GSEA2. These results suggested that the promotion of the chemosensitivity by PPARG might also be through the regulation of cell motivation related pathways.

## 4. Discussion

PPARG has been shown to increase the chemosensitivity in several human carcinomas, including nonsmall-cell lung carcinoma, breast carcinoma, and pancreatic carcinoma [4–6]. Here, we tested the potential role of increased PPARG expression in the chemosensitivity in HSCC, integrating literature-based pathway analysis and expression data analysis. Our results showed that PPARG plays roles in both chemosensitivity-inhibiting and promoting pathways and could improve the chemosensitivity through the positive regulation of cell proliferation-related pathways and the negative regulation of cell motility-related pathways.

Literature-based pathway analysis first identified seven chemosensitivity promoters that could be stimulated by increased PPARG expression, as shown in Figure 1. Each of the relations identified in Figure 2 was supported by at least one scientific report. For instance, Takeda et al. showed that the activation of PPARG increases the expression of BMP6 and BMP7 [11], while the expression of BMP6 was positively related to the chemosensitivity of breast cancer [12]. Chuang et al. showed that PPARG could directly increase the expression of NME1 [13], and NME1 increases the chemosensitivity of multiple squamous carcinoma cells [14, 15]. More information about the pathways in Figure 2 can be found in PPARG_HSCC: Regulatory_Pathway1. Expression analysis showed that these PPARG-driven chemosensitivity promoters were all upregulated in the HSCC CSP group compared with the CSNP group, which provides supports for the literature-based pathway. In addition, GSEA results showed that these PPARG-driven chemosensitivity promoters were mainly enriched within the cell proliferation-related pathways (Table 1), indicating that PPARG may influence the chemosensitivity through the regulation of carcinoma-related cell proliferation pathways.

**Figure 3:**
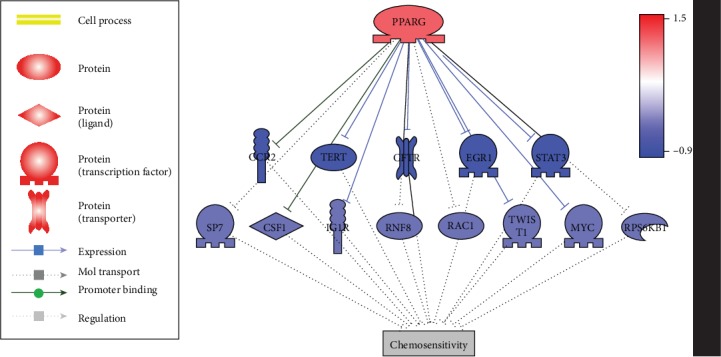
The chemosensitivity-inhibiting pathway built through literature data analysis and tested by HSCC expression data. The blue color of genes represents decreased expression in the chemosensitive group compared with the nonchemosensitive group.

On the other hand, literature-based pathway analysis also identified 13 chemosensitivity inhibitors that were suppressed by the activation of PPARG (Figure 2). For example, TERT was negatively correlated with chemosensitivity of hepatocellular carcinoma, bladder cancer, and head and neck cancers [16–18]. Ogawa et al. showed that PPARG ligands inhibit TERT expression through a receptor-dependent suppression of the TERT promoter [19]. Therefore, increased PPARG expression may indirectly activate chemosensitivity by suppressing its inhibitor. More pathways can be found in PPARG_HSCC→Regulatory_Pathway2. The expression of these PPARG-suppressed molecules in Figure 3 demonstrated the downregulated expressions in the HSCC CSP group, which supports the literature-based pathways for the chemosensitivity in HSCC. GSEA results showed that these molecules in Figure 3 mainly play roles in the cell motility and locomotion-related pathways (Table 2),suggesting that PPARG may also activate the chemosensitivity through the regulation of cell motivation-related pathways. Cell motivation and cell proliferation have been well connected with the etiology and prognoses of HSCC [20]. However, so far, little knowledge has been suggested regarding how cell proliferation and motivation were linked to chemosensitivity. The pathway we built and the GSEA revealed pathways indicated that the genes regulating cell proliferation and motivation might also play roles in the chemosensitivity regulation.

Expression analysis showed that PPARG demonstrated increased expression levels in CSP group (LFC = 0.50). However, due to the limited sample size, the expression level difference of PPARG between did reach statistical significance (*p* value = 0.25). Further study using larger datasets should be conducted to test the relation between PPARG expression and chemosensitivity in HSCC.

## 5. Conclusion

Our results identified potential pathways suggesting the chemosensitivity-promoting role of PPARG in HSCC cells, which may through the regulation of cell proliferation and motility-related pathways. Further studies with large datasets were guaranteed for the test of PPARG-chemosensitivity relation in HSCC.

## Figures and Tables

**Table 1:** The top 10 GO terms enriched with the eight genes from the chemosensitivity-promoting pathway.

GO ID	Name	# of entities	Overlap	Overlapping entities	*p* value
0048662	GO: negative regulation of smooth muscle cell proliferation	82	4	PPARG; TP53; MIR145; PTEN	0.00037
0010660	GO: regulation of muscle cell apoptotic process	125	4	PPARG; TP53; MIR145; PTEN	0.0010
0090200	GO: positive regulation of release of cytochrome c from mitochondria	33	3	BAX; TNFSF10; TP53	0.0014
0030162	GO: regulation of proteolysis	997	6	PPARG; BAX; PTEN; SERPINB5; TNFSF10; TP53	0.0014
0048147	GO: negative regulation of fibroblast proliferation	40	3	BAX; PPARG; TP53	0.0020
2001235	GO: positive regulation of apoptotic signaling pathway	213	4	BAX; PTEN; TP53; TNFSF10	0.0022
0010661	GO: positive regulation of muscle cell apoptotic process	48	3	PPARG; TP53; PTEN	0.0022
0048660	GO: regulation of smooth muscle cell proliferation	215	4	PPARG; MIR145; PTEN; TP53	0.0022
0090199	GO: regulation of release of cytochrome c from mitochondria	53	3	TP53; BAX; TNFSF10	0.0024
0090403	GO: oxidative stress-induced premature senescence	4	2	TP53; ARNTL	0.0024

**Table 2:** The top 10 GO terms enriched with the 13 genes from the chemosensitivity- inhibiting pathway.

GO ID	Name	# Of entities	Overlap	Overlapping entities	*p* value
2000147	GO: positive regulation of cell motility	630	9	CSF1; IGF1R; TWIST1; CCR2; RPS6KB1; EGR1; TERT; RAC1; MYC	2.37E-07
0051272	GO: positive regulation of cellular component movement	650	9	CSF1; IGF1R; TWIST1; CCR2; RPS6KB1; EGR1; TERT; RAC1; MYC	2.37E-07
0040017	GO: positive regulation of locomotion	666	9	CSF1; IGF1R; TWIST1; CCR2; RPS6KB1; EGR1; TERT; RAC1; MYC	2.37E-07
0030335	GO: positive regulation of cell migration	603	8	CSF1; IGF1R; CCR2; RPS6KB1; EGR1; TERT; RAC1; MYC	4.29E-06
0048660	GO: regulation of smooth muscle cell proliferation	215	6	IGF1R; PPARG; RPS6KB1; EGR1; TERT; MYC	1.13E-05
0071453	GO: cellular response to oxygen levels	221	6	PPARG; TWIST1; CFTR; EGR1; TERT; MYC	1.13E-05
0097305	GO: response to alcohol	460	7	PPARG; STAT3; IGF1R; RPS6KB1; CFTR; EGR1; MYC	1.69E-05
0043434	GO: response to peptide hormone	479	7	PPARG; STAT3; IGF1R; RPS6KB1; CFTR; EGR1; MYC	1.96E-05
0032870	GO: cellular response to hormone stimulus	520	7	PPARG; STAT3; IGF1R; RPS6KB1; CFTR; EGR1; MYC	3.07E-05
0048661	GO: positive regulation of smooth muscle cell proliferation	130	5	IGF1R; EGR1; TERT; MYC; RPS6KB1	3.49E-05

## Data Availability

The data in our study are available from the corresponding author upon reasonable request.

## Supplementary Materials

Supplementary Materials PPARG_HSCC: detailed results of pathway analysis and mega-analysis for PPARG and HSCC relationship study.
